# Improving the prediction of bitumen’s density and thermal expansion by optimizing artificial neural networks with Optuna and TensorFlow^☆^

**DOI:** 10.1016/j.mex.2025.103524

**Published:** 2025-07-30

**Authors:** Eli I. Assaf, Xueyan Liu, Sandra Erkens

**Affiliations:** a Delft University of Technology, Delft, the Netherlands; b Ministry of Infrastructure and Water Management (Rijkswaterstaat), the Netherlands

**Keywords:** Machine learning, Neural networks, Molecular dynamics, Optuna, TensorFlow

## Abstract

Previous work demonstrated that Random Forest Regressors (RFRs) could estimate the physical properties of bitumen using molecular descriptors derived from Molecular Dynamics (MD) simulations, thereby reducing the need for computationally intensive simulations. However, due to their decision-tree structure, RFRs lack true predictive capabilities, particularly for interpolation and extrapolation beyond the training data.

This study advances that foundation by employing Artificial Neural Networks (ANNs), which—when properly trained—can capture complex relationships with greater continuity and generalizability. Beyond simply replacing RFRs, we develop a fully automated framework for constructing Machine Learning Models (MLMs) to predict density and thermal expansion coefficients of bitumen. Using Optuna for hyperparameter optimization, we ensure that the information extracted from MD simulations is utilized effectively.

The resulting ANN models accurately reproduce MD-predicted densities, achieving R^2^>0.99, MSEs below 0.1 %, and maximum absolute errors below 5 % on test data. In addition to reducing computational cost, the models exhibit improved interpolation and extrapolation capabilities, enabling reliable predictions for properties, ranges, and compositions not explicitly simulated.

Key aspects of our approach include:

• Transitioning from RFRs to ANNs, improving generalization, interpolation, and predictive accuracy.
• Automated hyperparameter optimization, leveraging Optuna to maximize model efficiency.
• Expanding applicability, enabling property prediction for unseen compositions without additional MD simulations.

## Graphical abstract

**Figure:**
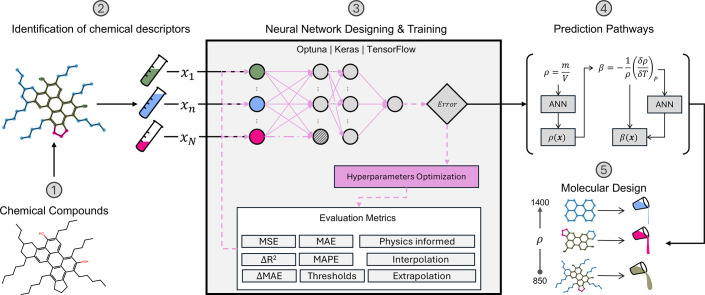

**Specifications table**

**Table:** 

**Subject area**	Chemistry
**More specific subject area**	Material property prediction / Machine Learning / Molecular Dynamics
**Name of your method**	Design and Optimization of Neural Networks
**Name and reference of original method**	E.I. Assaf, X. Liu, P. Lin, S. Ren, S. Erkens, Predicting the properties of bitumen using machine learning models trained with force field atom types and molecular dynamics simulations, Materials & Design 246 (2024) 113,327 https://doi.org/10.1016/j.matdes.2024.113327. E.I. Assaf, X. Liu, P. Lin, S. Ren, S. Erkens, Predicting the diffusion coefficients of rejuvenators into bitumens using molecular dynamics, machine learning, and force field atom types, Materials & Design 248 (2024) 113,502. https://doi.org/10.1016/j.matdes.2024.113502
**Resource availability**	Supplementary Information

## Background

Bitumen, a complex hydrocarbon material derived from petroleum distillation, plays a crucial role in infrastructure applications such as road construction and roofing [1]. With global production exceeding 100 million tons annually, understanding its physical behaviour under varying environmental conditions is essential for ensuring long-term durability and performance [2]. However, bitumen’s chemical composition is highly diverse and evolves due to variations in crude oil sources, refining processes, and the incorporation of additives or recycled materials [3]. This variability complicates efforts to correlate its molecular structure with macroscopic properties.

Traditional analytical techniques, such as Saturates, Aromatics, Resins, and Asphaltenes (SARA) fractionation, Fourier Transform Infrared Spectroscopy (FTIR), and elemental analysis, provide valuable insights into bitumen composition but do not fully capture its molecular heterogeneity [4,5]. Empirical and semi-empirical approaches, including thermodynamic models [[6], [7], [8]] and quantitative structure-property relationships (QSPR) [9], attempt to correlate composition with macroscopic properties, yet they struggle with the high dimensionality and nonlinear nature of bitumen’s physicochemical nature. Molecular Dynamics (MD) simulations offer a more fundamental approach, allowing for the computation of material properties from first principles [10]. However, their high computational cost and limited scalability present significant challenges when applied to complex hydrocarbon systems over extended timescales [11].

To overcome these limitations, Machine Learning Models (MLMs) methods have emerged as a promising alternative for property prediction [[12], [13], [14]]. These enable the extraction of meaningful correlations between molecular descriptors and computationally observed material properties derived from MD simulations. Prior work by our group explored the use of Random Forest Regressors (RFRs) [15] for this purpose, demonstrating their capability to predict physical properties such as densities, heat capacities, and thermal expansion coefficients [16,17]. Although decision tree-based models such as Random Forest Regressors (RFRs) are computationally efficient and easy to implement, they lack the capacity to interpolate smoothly between data points and can yield discontinuous outputs. These limitations make them less suitable for tasks that require continuous, physically consistent predictions, such as the estimation of thermophysical properties [18].

To improve predictive accuracy and model generalization, this study focuses on the development and optimization of Artificial Neural Networks (ANNs) [19] for the prediction of bitumen properties – namely densities and thermal expansion coefficients. Using the same dataset of molecular descriptors and observed properties as in our previous work, this research implements an automated hyperparameter tuning framework based on Optuna [20], integrated with Keras/TensorFlow [21,22] and Scikit-learn [23]. By systematically exploring a well-defined parameter space, the optimized ANNs aim to achieve superior predictive performance while minimizing the risk of overfitting or underperforming configurations – limitations identified in the RFR models employed in our previous publications. The following sections describe the methodological framework used to achieve this objective.

## Method details

This study employs an automated framework to optimize ANNs for predicting densities and thermal expansion coefficients obtained from MD simulations. Using Optuna for optimization alongside TensorFlow and Scikit-learn, the framework systematically tunes architectures and hyperparameters to balance predictive accuracy and computational efficiency. Executed on DelftBlue [24], Optuna performs iterative hyperparameter optimization over 2048 trial cases for each case study, refining models according to guiding functions and statistical criteria. The best-performing configurations are retrained on the full dataset before predicting the density of bitumens across varying conditions, such as temperature.

**Fig. 1:**
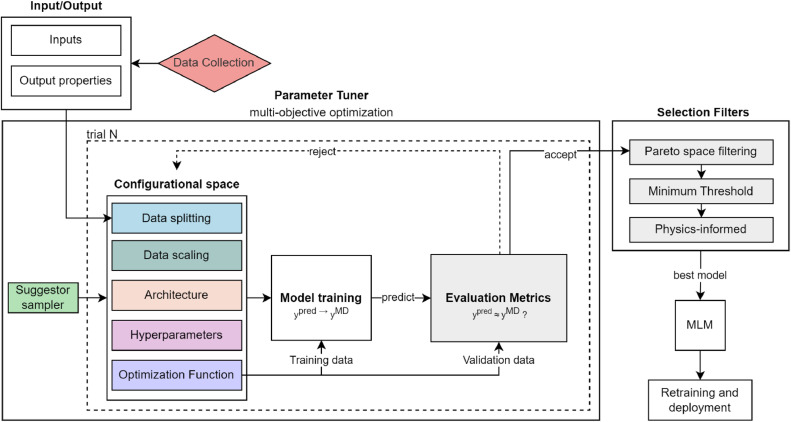
Diagram depicting the workflow for designing and training the ANNs of this study. Coloured blocks correspond to a subsection in this study, detailing its role and associated descriptions in the training and optimization process.

A schematic representation of the workflow is shown in Fig. 1. The workflow consists of eight primary components, each of which influences the final model's predictive capability and performance. Six of these components, highlighted in colour, correspond to tuneable aspects of the modelling process – conventionally subject to tuning in the literature [[25], [26], [27]]: (1) Suggester Sampler, (2) Data Splitting, (3) Data Scaling, (4) Model Architecture, (5) Hyperparameters, and (6) Optimization (i.e., loss) Function.

The Data Collection component captures the details involved in generating the inputs/outputs data used to train the models. The Evaluation Metrics component (and along with them, Selection Filters), serves as the criteria for assessing model performance across different trials. Although not part of the tuning process, these are responsible for selecting the best model from the top performing models found by Optuna. These models often form a Pareto-optimal front [28], requiring more advanced selection techniques to identify the best performer. Fig. 7 summarizes the design space sampled by Optuna to generate different trial cases.

The following subsections provide a detailed description of each stage in the tuning workflow – including the selection of Inputs and Outputs - explaining the rationale behind design choices and their role in improving model performance.

**Data Collection.** This study uses the dataset previously published in [16], which was generated through Molecular Dynamics simulations across a range of bituminous samples and temperatures. A brief overview of the setup is provided below, along with a construct of the resulting dataset used to train the models of this manuscript.

The work in focused on a collection of bitumen samples and structurally similar compounds, all belonging to the same broader hydrocarbon family—namely, amorphous mixtures that transition between liquid- and solid-like behaviour within the temperature range selected. In total, three different bitumen sources were selected and subjected to five distinct aging degrees, corresponding to fresh, short-term aged, and three levels of long-term aging (labelled long, longer, and longest). These were also blended with additives or rejuvenators commonly used in industry. The purpose of this was to expand the chemical diversity of the dataset and to enable detection of systematic relationships between chemical structure and bulk physical properties relevant to practical applications in the bitumen industry. Given the difficulty of achieving such a level of systematic variation through experimental work alone, Molecular Dynamics simulations were used in this study as a computational alternative.

Altogether, 197 bitumen-based systems—varying in origin, aging level, and additive content—were translated into molecular models for simulation. The molecular structures forming the basis of these models were taken from Greenfield [29] and Shisong [30], which are widely used and validated in MD studies concerning bitumen. Each model was assembled by selecting molecules from this set and packing them into a mixture containing approximately 6000 atoms (corresponding to roughly 80 molecules per sample). The composition of each system was tuned to match available experimental data, including SARA fraction distributions, elemental analysis, gel permeation chromatography (GPC) profiles, and, where available, FTIR spectra.

Once constructed, each system was subjected to a virtual temperature sweep, generating configurations at nine discrete temperatures, namely −60, −20, 0, 25, 60, 120, 135, 160, and 200 °C. Each simulation at a given temperature involved a sequence of four steps: Heating, Equilibration, Extraction, and Stability Assessment. The dataset used in this study was built from the configuration at the end of the Extraction phase, which consists of a 5-nanosecond-long NPT ensemble run designed to capture the equilibrium dynamics of the system at the target temperature. The Stability phase, of evaluative nature and excluded from property extraction, involved NVT and NVE runs without external temperature or pressure control to evaluate the system's intrinsic stability to such conditions.

The Molecular Dynamics simulations are conducted using LAMMPS, employing the Polymer Covalent Force Field (PCFF) [31] to define both intra- and interatomic interactions, which are necessary for evaluating forces and energies throughout the simulation. Atomic motion is integrated using the modified Nosé–Hoover thermostat [32], as implemented in LAMMPS via the standard **fix npt** command, which includes a velocity drag factor to reduce oscillatory behaviour. Both temperature and pressure damping parameters are set to 500 timesteps, and the velocity drag coefficient is fixed at 1.0. Simulations are carried out at a pressure of 101,325 Pa, using a timestep of 0.5 fs.

The final dataset comprises 1773 distinct configurations, each chemically and/or conditionally unique. Molecular structures were initially constructed using **SMI2PDB** [33] and converted into LAMMPS-compatible input formats via **PDB2DAT** [34]. The preparation of LAMMPS scripts and execution of the simulations were performed using Scymol [35]. Further details on the simulation setup and data processing can be found in the Supplementary Information of [16]. This includes a complete start-to-end script that initializes a molecular system and produces the output data necessary to reproduce a chemical system across all temperatures.

The simulations were conducted on the DelftBlue supercomputer, employing 16 cores of Intel XEON E5–6248R processors with 1 GB of memory allocated per core. Generating one additional input entry, such as a sample at a different temperature, required approximately three hours of computation, while producing a chemically new sample across all temperatures took about 24 h.

**Inputs Selection.** The input parameters utilized to train the ANNs in this study are derived from the chemical composition of the samples (inputs $x_{1}$ through $x_{30}$), molecular mass ($x_{31}$), and temperature ($x_{32}$). The chemical composition is represented as a vector of 30 values, each indicating the fractional content (by number) of a specific atom type in the sample, given by Eq. (1), as follows:

(1) $$a_{i,\;1-30}=\frac{1}{N}\left(\sum a_{1},\sum a_{2},\ldots ,\sum a_{30}\right).$$

where N is the total number of atoms, and $a_{i}$ corresponds to the number of a certain atom type in the sample. File */atom_type_formulas.xlsx* in the Supplementary Information displays the “atom-type” formula ($a_{i,\;1-30}$) for all the molecular models used in this study.

**Fig. 2:**
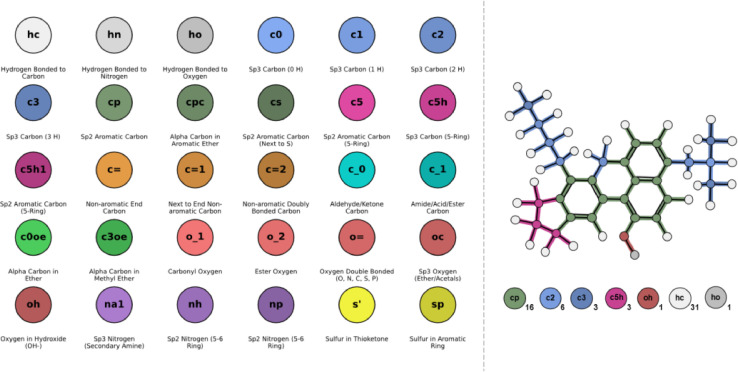
*(Left)* A list of atom types and their descriptions, derived from the PCFF force field. These atom types serve as the fundamental building blocks for formulating "atom type" formulas, which are used to chemically characterize MD systems and train ANNs. The atom types are categorized into ten broader functional groups, each represented by a distinct main colour, with individual atom types distinguished by varying shades within their assigned group. *(Right)* An example of atom type assignment for a randomly selected resinous molecule.

The atom types are identified – aligning with our previous studies -by combining all atom types determined using the PCFF force field across the studied samples, with detailed descriptions of each atom type provided in Fig. 2. The PCFF force field classifies atoms not only by their element but also by characteristics such as hybridization state, coordination, ring structure presence, aromaticity, and, in specific cases (e.g., sulphur atoms), the nature of their neighbouring atoms.

Utilizing an "atom-type" composition formula for each model, rather than a conventional chemical formula that groups atoms by elemental symbol, enables the differentiation of chemically distinct atoms and their functional roles. This approach provides a comprehensive depiction of the sample's topological construct without necessitating direct visualization of its chemical structures.

**Fig. 3:**
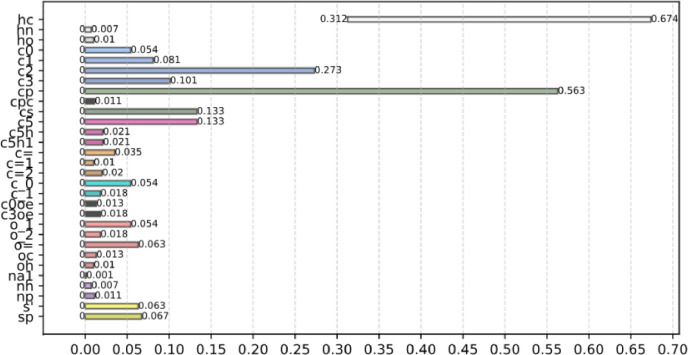
Number fraction ranges for all atom types in the data set of molecular systems utilised.

Fig. 3 shows the number fraction ranges of all atom types present in the dataset, providing an overview of the chemical diversity covered by the samples used in this study.

Input $x_{31}$, representing the sample's average molecular mass, accounts for molecule size in the molecular model. It introduces size-dependency to avoid cases where $x_{1}$ through $x_{30}$ fall within valid bounds but correspond to molecules significantly smaller or larger than those in the reference dataset. The values of $x_{31}$ range from 200 g/mol to 1000 g/mol, encompassing the molecular masses common in bituminous materials [39]. The values of $x_{32}$, representing temperature, range from −60 °C to 200 °C, encompassing the majority of thermal conditions encountered by bitumen during its lifecycle, from production to its application in pavements.

As a result, the Inputs x for each compound are defined as 32-element vectors containing the atom type formulas of each system, average molecular weight ($m_{w}$), and temperature (T), in that order, as shown in Eq. (2):

(2) $$x=\left(a_{1},a_{2},\ldots ,a_{n},\ldots a_{30},\;m_{w},\;T\right)$$

**Output Properties Selection.** The outputs correspond to the properties targeted by the ANNs. While a total of 12 properties is reported in [16]—ranging from fundamental MD quantities such as potential energies (e.g., total, van der Waals) to practical thermophysical properties like density and heat capacity—this manuscript focuses only on two: Density (ρ) and Thermal Expansion Coefficient (β). This restriction simplifies the scope of this manuscript without limiting the applicability of the methodology, which remains extensible to all other properties in the original dataset.

Density is chosen due to its fundamental character, predictable dependence on temperature, and direct connection to intermolecular interactions and volumetric behaviour. It serves as a proxy for related properties, including molar volume and thermal expansion, and is well-suited for assessing the predictive power of the machine learning model across structurally diverse systems—from small hydrocarbons to large asphaltenes.

The ANNs are trained to predict both ρ and β, defined in (3), (4), respectively:

(3) $$\rho =\frac{m}{V}$$

and

(4) $$\beta =-\frac{1}{\rho }{\left(\frac{\delta \rho }{\delta T}\right)}_{P}$$

The goal is to approximate these properties ($y_{i}$) with high accuracy relative to MD predictions ($y_{i}^{MD}$). Each ANN model is described in Eq. (6) as:

(5) $$ANN_{i}=f{\left(x\right)}_{i}=f\left(a_{1},a_{2},\ldots ,a_{n},\ldots a_{42},\;m_{w},\;T\right)=y_{i}\approx y_{i}^{MD}$$

where $x_{i}$ denotes the input features representing the molecular system, and $y_{i}$ is the target property to be predicted.

**Suggester Sampler.** The Suggester Sampler is an essential component for exploring a diverse range of parameter configurations in an optimization framework. Its primary function is to propose new parameter values based on predefined distributions, ensuring that the search space is thoroughly and evenly sampled. Three types of samplers, including **TPESampler** (Tree-structured Parzen Estimator), **NSGAIISampler** (Nondominated Sorting Genetic Algorithm II), and a **GridSampler**, are integrated into the optimization process detailed in this manuscript. These samplers are native to Optuna and are fully compatible with studies involving multiple objectives, as well as supporting the exploration of both numerical (float or integer alike) and categorical parameters concurrently.

**Data Scaling.** Data scalers are essential for centring and standardizing numerical data, minimizing numerical errors and ensuring consistency in how ANNs process the inputs vector. Since MLM algorithms are sensitive to data scaling and distribution, the choice of scaler can significantly impact learning and performance [27]. To address this, three widely used scalers—**MinMaxScaler, StandardScaler**, and **MaxAbsScaler**—are incorporated into the optimization process detailed in this manuscript (Table 1). The optimization process includes the random categorical selection of scalers for both inputs and outputs, allowing for the exploration of both uniform and mixed scaler combinations (e.g., inputs scaled using MinMaxScaler and outputs using StandardScaler).

**Table 1:** Table of data scalers, including their mathematical formulations and descriptions, available for categorical selection during the optimization process for scaling inputs and outputs.

Scaler	Formula	Description
MinMaxScaler	$x^{\prime }=\frac{x-\text{min}(x)}{\max(x)-\text{min}(x)}$	Scales inputs to a specified range (default: [0, 1]), preserving relative relationships between input values.
StandardScaler	$x^{\prime }=\frac{x-\mu }{\sigma }$	Centres inputs around zero with unit variance, ensuring a standard normal distribution.
MaxAbsScaler	$x^{\prime }=\frac{x}{\text{max}(\left|x\right|)}$	The MaxAbsScaler scales data to [−1,1] by dividing by the maximum absolute value, preserving sparsity.

**Data Splitting.** Splitting data into *training* and *testing* sets is essential for evaluating MLMs. Training data enables the model to learn patterns, while testing data assesses its performance on unseen data. Using 100 % of the data for training may improve the model's fit to observed data but prevents reliable performance evaluation due to the absence of a testing set. A balance is necessary: larger training sets enhance learning, while larger testing sets ensure stable and representative evaluation metrics [36]. To explore this trade-off, the manuscript examines training-to-testing ratios ranging from 70/30 to 90/10, in increments of 5 %, assessing their impact on model learning and evaluation. These ratios are commonly used in training MLMs. While adopting a standard 80/20 split could simplify the optimization process by reducing the dimensionality of the search, understanding how models respond to varying amounts of available data is crucial for determining whether the optimization process is not limited by the training dataset size [37].

**Machine Learning Model Architectures.** This study explores three types of ANNs, treating the choice of architecture as a hyperparameter to be optimized. The primary distinction among these architectures lies in their learning mechanisms and the flow of input information through the network to generate outputs [38]. The architectures examined include a Fully Connected Neural Network (FCNN), a Wide and Deep Neural Network (WDNN), and a Residual Neural Network (ResNet), which are detailed in the following sections.

**Fig. 4:**
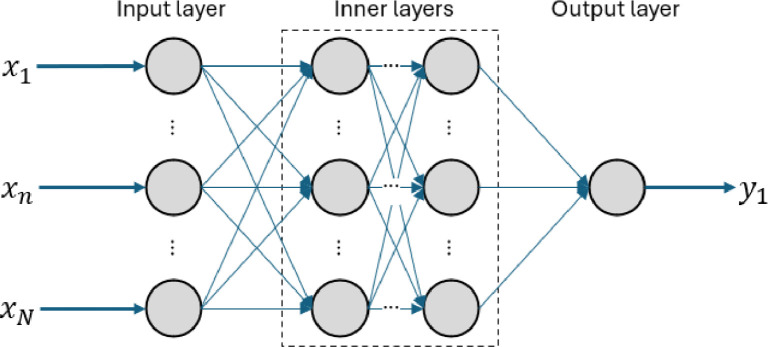
Illustration of a Fully Connected Neural Network (FCNN) architecture, consisting of sequentially connected layers where each layer is fully connected to the next.
**2) Wide and Deep Neural Network (WDNN).** The WDNN extends the traditional approach of a FCNN by introducing two separate pathways for processing information: a *wide* pathway and a *deep* pathway [40]. The wide pathway connects the input directly to the output through a simple linear transformation, which helps retain more evident relationships between inputs and target outputs. The deep pathway, on the other hand, passes the input through multiple dense layers, allowing the network to extract more complex and abstract patterns. At the end of these pathways, the outputs from both are combined into a single representation, which is processed by a final layer. This architecture is depicted in Fig. 5.

**1) Fully Connected Neural Network (FCNN).** The FCNN is a straightforward NN architecture consisting of sequentially interconnected layers where each layer is fully connected to the next [39]. This architecture is depicted in Fig. 4.

**Fig. 5:**
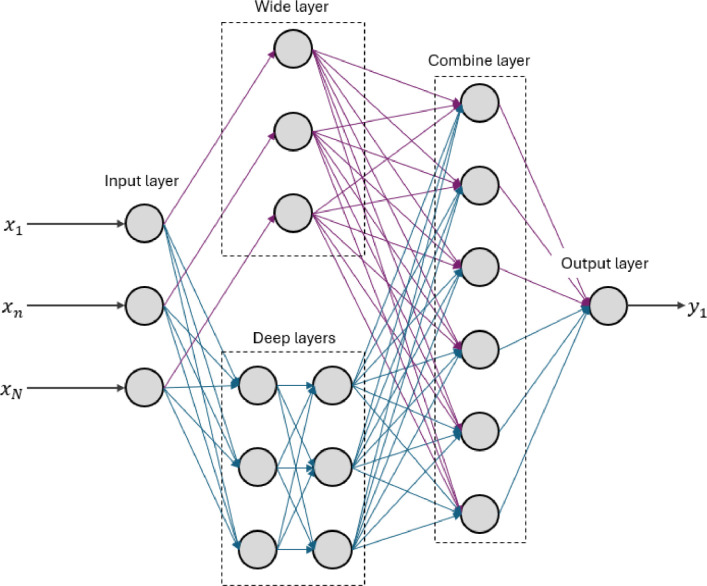
Illustration of a Wide and Deep Neural Network (WDNN) architecture, featuring two distinct pathways: a wide pathway (shown in purple) for capturing simpler linear relationships and a deep pathway (shown in blue) for modelling complex, non-linear patterns. The outputs of both pathways are combined and processed by a final dense layer to generate the prediction.
**3) Residual Neural Network (ResNet).** The ResNet introduces an architecture where the output of certain layers is directly added to the output of deeper, non-adjacent layers [41]. Skipping adjacent connections ensures that some information bypasses intermediate transformations, helping the network preserve and reuse important inputs caught by earlier nodes, preventing degradation of important information as the information reaches the ANN’s output. This design is particularly effective in deeper network configurations with multiple inputs but where a handful of inputs hold clear influence over the output, expected in the study of physical properties. This architecture is depicted in Fig. 6.

**Fig. 6:**
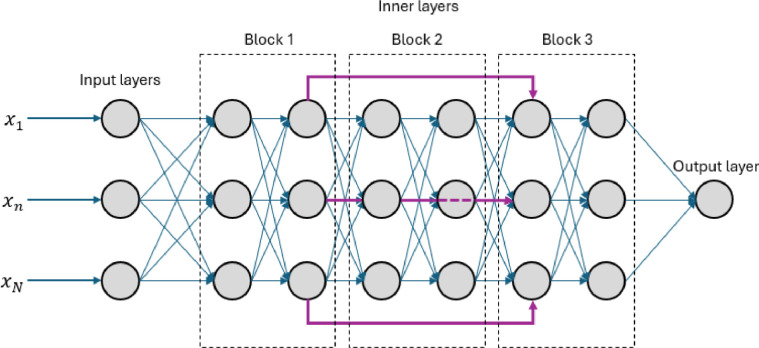
Illustration of a Residual Neural Network (ResNet) architecture, where skip connections (highlighted in purple) allow the output of earlier layers to bypass intermediate transformations and connect directly to deeper layers.

**Hyperparameters.** Hyperparameters define the structure of an ANN and govern the training process. Unlike model parameters—such as weights and biases—that are learned during training, hyperparameters are set beforehand and influence aspects like an ANN’s depth, learning rate, and optimization strategy [27]. Their selection significantly impacts model performance. Optuna is used to systematically search for configurations that yield the most accurate predictions.

**Fig. 7:**
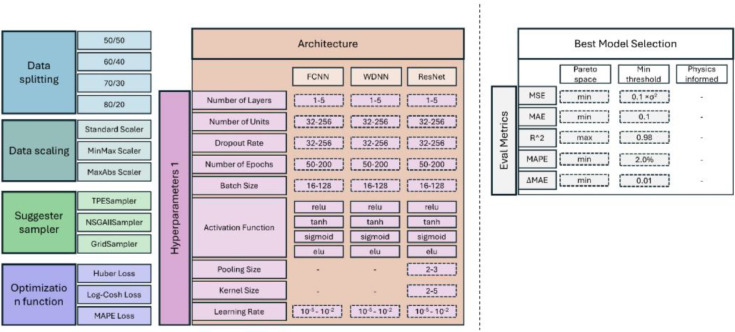
Overview of the optimization space explored by Optuna for generating an ANN using the workflow of Fig. 1.Solid-line boxes represent categorical choices, while dashed-line boxes indicate continuous parameters sampled within specified ranges.

While many hyperparameters are common across the selected ANN architectures—namely FCNN, WDNN, and ResNN —some are specific to these architectures. For example, a hyperparameter affecting only the “wide” section of a WDNN does not apply to other architectures. A complete list of hyperparameters (and all other tuneable parameters), along with their descriptions and optimization ranges to be studied by Optuna, is provided in Fig. 7.

**Model Evaluation Techniques.** The evaluation of an ANN’s performance occurs both during training and post-training stages. During training, Loss Functions guide the model by penalizing errors (e.g., minimizing mean squared error). Post-training, the model's performance is assessed using Performance Evaluation Metrics. The validation methods for both stages are detailed in the following sections.

**Loss Function.** A loss function measures the discrepancy between predicted and true values, guiding the model toward improved accuracy. In models predicting physical properties, selecting an appropriate loss function is crucial to ensure adherence to fundamental trends, such as the monotonic decrease in density with increasing temperature, while minimizing errors in alignment with these principles. In this manuscript, the loss function is treated as a hyperparameter, exploring three widely used metrics—**Huber Loss** ($L^{Hubber}$), **Log-Cosh Loss** ($L^{Log-Cosh}$), and **Mean Squared Error Loss** ($L^{MSE}$) — all natively available in TensorFlow/Keras. Details of these metrics are summarized in Table 2. Additionally, the δ parameter in the Huber Loss expression is optimized as a hyperparameter, with values ranging from 0.1, increasing sensitivity to outliers, to 10, reducing this sensitivity for greater overall robustness.

**Table 2:** Description of the three Loss metrics, treated as hyperparameters, utilized by the loss function to guide Optuna during model training.

Loss Metric	Expression	Description
Huber Loss ($L^{Hubber}$)	$L^{Hubber}=\{\begin{array}{c} \frac{1}{2}{\left(y_{MD}-y\right)}^{2}\;if\;\left|y_{MD}-y\right|<\delta \\ \delta |y_{MD}-y|-\frac{1}{2}\delta^{2}\;if\;\left|y_{MD}-y\right|>\delta \end{array}$	Balances sensitivity to small errors with robustness to outliers
Log-Cosh Loss ($L^{Log-Cosh}$)	$L^{Log-Cosh}=\text{log}\left(\text{cosh}(y_{MD}-y)\right)$	Encourages smooth predictions while being less sensitive to extreme deviations
Mean Squared Error Loss ($L^{MSE}$)	$L^{MSE}=\frac{1}{N}\sum {\left(y_{MD,i}-y_{i}\right)}^{2}$	Ignores small accumulating errors but overemphasizes outliers.

While stricter physically informed loss metrics, such as those used in [42,43], could be implemented, they require a clear understanding of the underlying relationships between inputs and outputs. Since most of the inputs in this study are derived from molecular topologies, which capture the connectivity and structural arrangement of atoms within a molecule, achieving such an understanding is challenging, even for density. Instead, the exploration of different, but conventional loss metrics (be it $L^{Hubber}$, $L^{Log-Cosh}$, or $L^{MSE}$) provides a practical balance between physical realism and general applicability.

**Performance Evaluation Metrics.** Performance evaluation is conducted through a systematic three-step process to identify the best-performing ANN among the hundreds generated during the tuning stage. The evaluation relies on five metrics: Normalized Mean Squared Error (MSE), Normalized Mean Absolute Error (MAE), Coefficient of Determination ($R^{2}$), Normalized Maximum Absolute Error (MaxAE), and Delta Coefficient of Determination ($\Delta R^{2}$). These metrics are defined as objectives in the Optuna study, where MSE, MAE, MaxAE, and $\Delta R^{2}$ are minimized, and $R^{2}$ is maximized, and details of their computation can be found in Table 2. All five metrics are treated with equal importance during the optimization and evaluation process.

Evaluation metrics are computed for both the training and testing subsets of the dataset (see Splitting). However, all final model assessments are based solely on predictions obtained from the testing set. This ensures that the reported metrics reflect model performance on previously unobserved data, thereby avoiding misleading evaluations that could arise from overfitting or memorization—issues that may occur when models are poorly optimized or inadequately regularized.

The process of filtering and selecting the "best" model among the multiple ones trained involves three steps, outlined below.

**Step 1: Pareto Solution Filtering.** The process begins by using Optuna to identify models that achieve a balance across multiple performance metrics, forming a set of Pareto-optimal solutions. A model is Pareto-optimal if no other model outperforms it across all metrics simultaneously, ensuring that gains in one metric do not come at a significant cost to others [44]. This approach yields a reduced set of models that represent the best compromises among the five objectives, while models outside the Pareto-optimal space are discarded.

Moreover, while the filtered Pareto-optimal solutions suggest good model performance based on the metrics of Table 3, hyperparameter selection can reveal underlying issues. During tuning via Optuna, ranking hyperparameter importance helps assess model robustness. A well-structured ANN should distribute influence among key hyperparameters rather than being dominated by one (>60 %), which would indicate inadequate exploration of the design space [45]. Additionally, the selected hyperparameters should align with established trends in literature to ensure meaningful tuning. If these criteria are not met, the model is likely to be overfit or poorly optimized despite favourable statistical metrics and is therefore discarded.

**Step 2: Static Threshold Filtering.** The Pareto-optimal models are then filtered based on predefined thresholds for each metric - detailed in Table 3. A model failing to meet any threshold is eliminated, ensuring that all retained models achieve a minimum acceptable level of performance across all metrics. Scale-dependent metrics (MSE, MAE, and MaxAE) are normalized against the range of the dataset, Δy=max(y)−min⁡(y)*.*
**Step 3: Additional Performance Metrics.** Even after Steps 1 and 2, it is still common for multiple ANNs to be considered as potential “best model solutions”. While some models may score slightly better in conventional statistical metrics (e.g., like those from Table 3), the differences can be negligible, making it difficult to justify selecting one over the others based solely on these scores. Moreover, certain models that perform well in these metrics may fail when evaluated against more specific, but physically relevant constraints. Therefore, Step 3 introduces an additional layer of evaluation, designed to refine the selection process by subjecting the best-performing models to a set of physically and field-relevant performance assessments. These additional metrics are detailed as follows:
**(a) Interpolative Performance.** MLMs should effectively interpolate between training data points, ensuring that predictions remain continuous, proportional, and physically reasonable. To evaluate interpolation, two approaches are employed, following the methods outlined by Belisle et Al [46]:
1. Pointwise Interpolation

**Table 3:** The model's performance is evaluated using five metrics optimized by Optuna, yielding a Pareto-optimal set where trade-offs exist between metrics. Models are then filtered by predefined thresholds, discarding those that fail to meet any, ensuring a minimum performance across all metrics.

Metric	Expression	Threshold
Normalized Mean Squared Error (MSE)	$MSE=\frac{1}{N}\sum_{i=1}^{N}{\left(\frac{y_{MD,i}-y_{i}}{\Delta y}\right)}^{2}$	MSE≤0.05
Normalized Mean Absolute Error (MAE)	$MAE=\frac{1}{N}\sum_{i=1}^{N}\left|\frac{y_{MD,i}-y_{i}}{\Delta y}\right|$	MAE≤0.05
Coefficient of Determination (R²)	$R^{2}=\sum_{i=1}^{N}\frac{{\left(y_{MD,i}-y_{i}\right)}^{2}}{{\left(y_{MD,i}-\bar{y}\right)}^{2}}$	R²≥0.99
Normalized Maximum Absolute Error (MaxAE)	$MaxAE=\begin{array}{c} max \end{array}\left(|\frac{y_{MD,i}-y_{i}}{\Delta y}{|}_{i=1}^{N}\right)$	MaxAE≤0.05
Delta Mean Absolute Error ($\Delta R^{2}$)	$\Delta MAE=|R_{train}^{2}-R_{test}^{2}|$	$\Delta R^{2}\leq 0.01$

The model is assessed only at existing test set points, ensuring that predictions at these locations follow smooth and physically meaningful trends. This guarantees that the model does not exhibit over-smoothing, excessive sensitivity, or trend violations when predicting values at known data points. The evaluation consists of three independent metrics.

First, artificial flattening is detected using Wavelet High-Frequency Energy [47], which quantifies the presence of rapid fluctuations in the predicted function, defined in Eq. (6) as:

(6) $$E_{w}=\sum_{i}c_{i}^{2}$$

where $c_{i}$ are the coefficients of the highest-frequency wavelet decomposition level of the predicted function. Models are accepted if their $E_{w}$ lies within the range of 0.005 to 0.03, which reflects smooth but non-flat trends consistent with physically realistic property variations.

Second, smooth and proportional transitions are analysed through the Total Variation Norm (TV), given in Eq. (7) as:

(7) $$TV=\sum_{i=1}^{N-1}\left|y\left(x_{i+1}\right)\right|-y\left(x_{i}\right)$$

where y(x) represents the model’s predicted value at x. Only models where 0.15<TV<15 are accepted – preventing cases abrupt changes or artificial smoothing dominate the trend.

Third, trend consistency is assessed through the Monotonicity Flips Count, defined in Eq. (8) as:

(8) $$M=\sum_{i=1}^{N-1}1\left[\left(y\left(x_{i+1}\right)-y\left(x_{i}\right)\right)\left(y\left(x_{i}\right)-y\left(x_{i-1}\right)\right)<0\right]\;$$

where M counts the number of trend reversals in the predictions. This metric highlights violations of physical monotonicity (e.g., density decreasing with increasing temperature). Models with M>2 are discarded.

2. Strict Interpolation:

In addition to testing at actual data points, the model is further evaluated on interpolated points generated between every consecutive test sample (i and j). Given two adjacent test points ($x_{i}$ and $x_{i+1}$), a set of 20 evenly spaced synthetic points is introduced in between using Eq. (9):

(9) $$x_{i,j}=x_{i}+j\frac{x_{i+1}+x_{i}\;}{20}$$

Predictions are computed at each synthetic point $x_{i,j}$, and the same three metrics—Wavelet Energy, TV-Norm, and Monotonicity Flips—are applied to evaluate smoothness, proportionality, and trend consistency between known data points.

Interpolation-based selection can be misleading when applied to models whose inputs have largely unknown effects on the target property (e.g., the impact of an etheric carbon of type “coe1” in a chemical system). However, this criterion is only used after models have passed all prior evaluation metrics. At this stage, the interpolation assessment does not determine a model’s fundamental validity but ranks well-performing models based on their ability to produce smoother and physically more reasonable predictions.

**(b) Extrapolative Performance.** MLMs should exhibit acceptable extrapolative performance, ensuring that their predictions remain physically reasonable even in scenarios beyond the training domain. To evaluate extrapolation, two approaches are employed:
1. Testing Beyond the Training Inputs Space:

This type of extrapolation is not inherently required, as the training already covers a broad inputs space. However, it is still desirable for a model to exhibit reasonable behaviour when applied to input values slightly beyond the training range. To assess this, input values are extended by 10 % beyond their original limits across 100 equally spaced points, and the **Interpolative Performance** method is applied to determine whether the model’s predictions remain physically plausible and follow reasonable trends. If extending the range limits by 10 % results in physically unreasonable conditions (e.g., predicting <0 % content of atom type “cp”), the test is omitted in such cases. The goal is not to achieve high accuracy in these regions but rather to identify models that collapse, become erratic, or deviate unreasonably when encountering values slightly outside their training regime.

2. Testing on Unseen Compounds

Although the dataset used to train the ANNs is deliberately partitioned to retain a portion of data for validation, it remains critical to assess model performance on entirely unseen chemical compounds. To simulate a realistic external-use scenario, the MLMs are applied to predict the properties of compounds that belong to the bituminous material family—such as mineral or synthetic oils, lubricants, industrial greases, and waxes. These materials share molecular similarities with bitumens, which are soft matter systems composed of high-molecular-mass hydrocarbons forming predominantly amorphous mixtures of liquids and solids. Notably, these extrapolative test compounds are completely excluded from the training and validation datasets, ensuring that the MLMs are truly tested in an unfamiliar predictive setting.

**Fig. 8:**
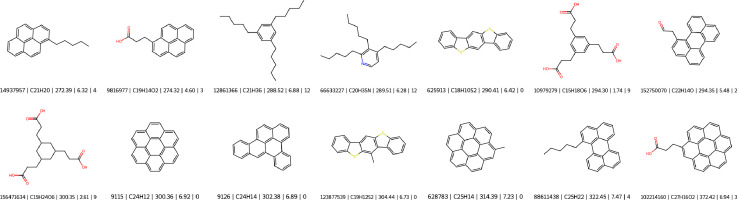
Collection of naturally occurring compounds, along with their PubChem ID, chemical formula, molecular weight, and polarity for testing the prediction performance of the ANNs.

For this validation process, a collection of 14 molecules was assembled by identifying structures in the PubChem [48] database with a Takimoto similarity index [49] comparable to that of the molecules in the study’s dataset. The presence of these molecules in PubChem provides evidence of their existence and natural occurrence, thereby supporting the practical applicability of the MLMs to realistically occurring compounds. Each selected molecule is treated as a separate system, representing a pure compound. These molecules are converted into MD models following the methodology outlined in **Data Collection** and in [16] and undergo identical simulation routines using Scymol. The physical properties obtained from these simulations are used to evaluate the performance of the MLMs. Fig. 8 provides a list of all benchmark molecules along with their corresponding PubChem IDs.

**(c) Predictive Pathway Comparison Check.** When a property can either be predicted directly using a dedicated MLM or derived from more fundamental predicted properties via a well-established equation, a comparison is performed to determine the more reliable approach. If the relationship governing the property is well-defined and its input parameters can also be predicted using MLMs, direct prediction may be unnecessary. Instead, models are first trained to predict the fundamental properties, and the target property is then computed from these predictions. If the computed values align with expected trends and show lower error propagation than direct predictions, the dedicated model for the derived property is discarded [50].

This issue is particularly relevant in physics-oriented fields, where many ML-predicted properties result from fundamental relationships. Ensuring that predictions remain physically consistent avoids redundancy, minimizes unnecessary model complexity, and reduces error propagation. However, if direct prediction outperforms the derived approach—such as when noise in fundamental property predictions amplifies errors or when the relationship between properties is not smoothly differentiable—the direct model is retained.

This evaluation provides a systematic framework for determining whether machine learning should be used to predict derived properties directly or if physical constraints should be enforced through indirect computation, as demonstrated by Zhao et al. [51]. For example, the thermal expansion coefficient (β) can be obtained in two ways: (1) by training an MLM to predict β, or (2) by first predicting density as a function of temperature, ρ(T), using an MLM, and then computing β using its fundamental definition presented in Eq. (4). The predictions remain subject to the constraints outlined in Interpolation and Extrapolation Performance.

**(e) Inference Speed and Practicality.** If multiple models remain after all previous checks, the final selection criterion is inference speed. Although not the primary determinant of model quality, a faster model is generally preferable, especially in this manuscript, where real-time or on-the-fly predictions are among the objectives. The model with the lowest inference time—while still satisfying all previous constraints—is chosen.

## Method validation

**Fig. 9:**
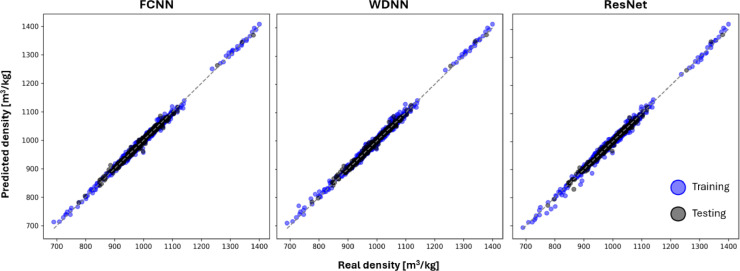
Scatter plots comparing predicted density values with MD-derived values for the best NN of each architecture. Blue dots represent training data predictions, while black dots indicate testing data, reflecting the MLM's true predictive capability. A perfect MLM would align along the 45-degree line, indicating no deviation between predicted and actual values.

The developed framework successfully employs ANNs to fully utilize the data presented in [16,17], moving away from the reliance on RFRs. ANNs were constructed to predict Density and Thermal Expansion Coefficients, achieving the same or superior evaluation metrics compared to our previous studies, even when comparing $r^{2}$ values alone. Fig. 9 illustrates how the best-performing FCNNs, WDNNs, and ResNets predict the density of hydrocarbons in both training and testing datasets. The corresponding evaluation metrics are reported in Table 4, with all three architectures meeting the constraints detailed in Table 3.

**Table 4:** Evaluation Metrics for the best NNs (of each architecture) designed and trained to compute Density.

Model	FCNN	WDNN	ResNet
MSE	0.000122	0.00011	0.000105
MAE	0.007756	0.007586	0.007252
MaxAE	0.06675	0.04592	0.05932
$R^{2}$	0.990129	0.990974	0.990839
$\Delta R^{2}$	0.004125	0.002083	0.001107

**Fig. 10:**
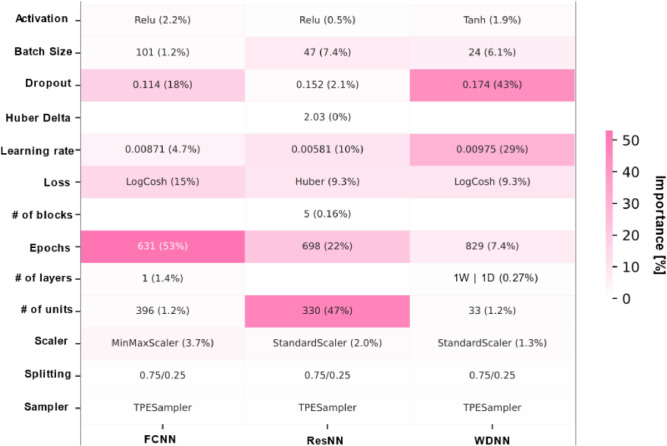
Optimal hyperparameters determined by Optuna for the best NNs of each architecture trained to predict density. Percentage values in parentheses indicate the relative importance of each hyperparameter as assessed by Optuna.

Fig. 10 summarizes the hyperparameter configurations for each architecture that yielded these results. Additionally, it provides insight into the relative influence of each tuned hyperparameter, expressed as a percentage in parentheses, based on Optuna’s optimization process. This information serves as a guideline for future researchers in identifying the most impactful tuning aspects. No single hyperparameter dominates the optimization process in an unnatural way, nor does any parameter assume extreme values that would result in excessive network complexity (e.g., an unreasonably large number of epochs). Instead, most hyperparameters remain well-balanced within their respective optimization ranges.

The best-performing model, based on a WDNN architecture, was selected due to its slightly superior effectiveness in predicting densities. This suggests that physical properties may benefit from model components that preserve clear trends from the initial training stages. Furthermore, the hyperparameter values for this model fall well within acceptable ranges, with all exerting an even influence during training. All trained ANNs achieve prediction times on the order of 100 milliseconds per compound, allowing near-instantaneous density estimation across architectures. Among these, the WDNN is the fastest, with an average prediction time of 33.4 milliseconds.

**Fig. 11:**
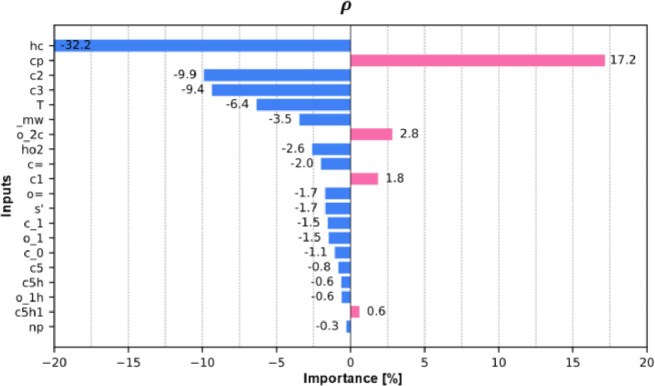
Bar plots showing the relative importance ( %) of the 20 most influential input features in predicting density. Magenta indicates positive contributions, while blue indicates negative ones. The plot reveals how each feature affects the predicted values. For instance, temperature strongly influences density (−6.4 %), where an increase lowers its value.

Fig. 11 shows bar plots of the relative importance (percentage) of input variables for predicting material density. This analysis identifies the features that most significantly affect density and indicates whether they contribute to its increase or decrease. This information helps researchers verify that the machine learning models capture physically consistent trends, such as the expected decrease in density with increasing temperature.

The top six contributors—atom types “hc”, “cp”, “c2”, and “c3”, along with temperature and molecular weight—collectively account for over 80 % of the model's explanatory power. Their respective contributions are −32 %, +17 %, +9.8 %, −9.3 %, −6.3 %, and −3 %. The remaining ∼20 % is primarily attributed to heteroatomic and polar carbon-based groups, such as carbonyls, which generally promote molecular cohesion and increase density.

The direction of the feature influences aligns well with established chemical understanding [52]. Atom types associated with reduced hydrocarbon features—such as “hc”, “c2”, “c3”, and other paraffinic or naphthenic groups—tend to lower density. Conversely, atom types linked to aromaticity, planarity, or polarity—such as “cp” and heteroatomic polar groups—are associated with higher densities due to enhanced stacking and stronger cohesive interactions.

Additionally, the relatively low importance attributed to temperature may initially seem counterintuitive, given the well-established decrease in density with increasing temperature. However, this should not be taken to imply that temperature plays a negligible role. Instead, the explanation lies in the composition of the dataset: densities range from approximately 715 to 1418 kg/m³, largely due to the broad chemical diversity of the compounds studied. In this context, structural differences account for most of the variance, thereby diminishing the relative contribution of temperature in the model's attribution.

## Example

**Fig. 12:**
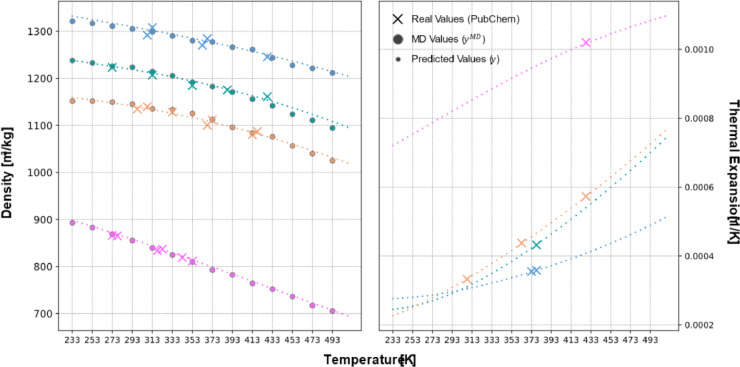
(Left) Density comparison for four novel molecules: obtained from PubChem (×), MD simulations (●), and ANN predictions (•). The predictions closely follow MD results while staying within the PubChem observations. (Right) Thermal expansion coefficient (β) computed from predicted density, ensuring smooth and physically consistent trends.

The plot in Fig. 12 (left) presents density-related data for four molecules from Fig. 8, all of which are entirely new structures—not included in the training or testing sets. It compares experimental density values retrieved from the PubChem ID database, densities obtained via MD simulations, and predictions made by the NN trained to estimate density. The results demonstrate that the NN reliably predicts density, closely matching the MD-derived values while remaining within a reasonable range of the PubChem reference data. Additionally, the NN successfully preserves monotonic interpolation between recorded temperature values.

## Limitations

Beyond the inherent constraints associated with ANNs, including their design and optimization challenges - it was observed that for physical properties that are inherently higher-order derivatives of more fundamental ones, it is generally more effective to train the ANN to predict the fundamental property and then compute the derived property using its governing physical equation. This is not due to any inherent limitation in the ANN’s ability to fit the derived property—direct predictions often yield values that align reasonably well with expected magnitudes—but rather due to the nature of how these properties are obtained. Derivative properties require values computed at infinitesimally close points, and when derived from scattered data points that are not sufficiently close in the differential sense, the resulting derivative curves can be highly erratic. This issue leads to predictions that, while providing a reasonable overall estimate, fail to capture smooth and physically consistent trends.

The thermal expansion coefficient (β), which, when extracted directly from MD simulations performed at large temperature intervals (>20 K), provides an accurate global estimate but results in a β(T) function that lacks smoothness, continuity, and monotonicity. Conversely, when density is predicted as a function of temperature, and β(T) is computed from its fundamental definition presented in Eq. (4), the resulting derivative curves are well-defined, yielding not only accurate values but also a function that is smooth, continuous, and physically realistic.

These findings emphasize the importance of evaluating each property individually to determine whether a direct prediction model or an indirect computation pathway is more appropriate. This distinction is illustrated in Fig. 12 (right), where the predicted β(T) for all compounds exhibits physically consistent behavior, reinforcing the necessity of selecting the optimal predictive pathway for each property.

## Ethics statements

This research did not involve human subjects, animal experiments, or data collected from social media platforms. No ethical approval was required.

## Declaration of generative AI and AI-assisted technologies in the writing process

During the preparation of this work the author(s) used OpenAI’s ChatGPT4o to shorten the length of certain sections. After using this tool/service, the author(s) reviewed and edited the content as needed and take(s) full responsibility for the content of the publication.

## Declaration of competing interest

The authors declare that they have no known competing financial interests or personal relationships that could have appeared to influence the work reported in this paper.

## Data Availability

Upon manuscript acceptance we will add the Machine Learning Models for use in the Supplementary Information

## Appendix Supplementary materials

Image, application 1
